# Identifying effects of genetic obesity exposure on leukocyte telomere length using Mendelian randomization

**DOI:** 10.7717/peerj.15085

**Published:** 2023-03-21

**Authors:** Bangbei Wan, Ning Ma, Cai Lv

**Affiliations:** 1 Department of Urology, Haikou Affiliated Hospital of Central South University, Xiangya School of Medicine, Haikou, Hainan, China; 2 Reproductive Medical Center, Hainan Women and Children’s Medical Center, Haikou, Hainan, China

**Keywords:** Body mass index, Telomere length, Body fat mass, GWAS, Single-nucleotide polymorphism

## Abstract

**Background:**

Observational studies have shown that obesity is closely associated with leukocyte telomere length (LTL). However, the causal relationship between obesity and LTL remains unclear. This study investigated the causal relationship between obesity and LTL through the Mendelian randomization approach.

**Materials and Methods:**

The genome-wide association study (GWAS) summary data of several studies on obesity-related traits with a sample size of more than 600,000 individuals were extracted from the UK Biobank cohort. The summary-level data of LTL-related GWAS (45 6,717 individuals) was obtained from the IEU Open GWAS database. An inverse-variance-weighted (IVW) algorithm was utilized as the primary MR analysis method. Sensitivity analyses were conducted *via* MR-Egger regression, IVW regression, leave-one-out test, MR-pleiotropy residual sum, and outlier methods.

**Results:**

High body mass index was correlated with a short LTL, and the odds ratio (OR) was 0.957 (95% confidence interval [CI] 0.942–0.973, *p* = 1.17E−07). The six body fat indexes (whole body fat mass, right leg fat mass, left leg fat mass, right arm fat mass, left arm fat mass, and trunk fat mass) were consistently inversely associated with LTL. Multiple statistical sensitive analysis approaches showed that the adverse effect of obesity on LTL was steady and dependable.

**Conclusion:**

The current study provided robust evidence supporting the causal assumption that genetically caused obesity is negatively associated with LTL. The findings may facilitate the formulation of persistent strategies for maintaining LTL.

## Introduction

Telomeres are DNA-protein complexes located on chromosome ends and play a critical role in guaranteeing the stability and integrality of chromosomes. An abnormal change in telomere length is closely related to human health. Leukocyte telomere length (LTL) is a quickly gauging indicator compared with other tissues. A previous study showed that LTL was closely related to the telomere length of tissues and served as an indicator that reflects telomere length in other tissues (Takubo et al., 2002). Mounting evidence has shown that extreme long LTL is strongly correlated with disease occurrence and development (Antwi & Petersen, 2018; Ayora et al., 2022; D’Mello et al., 2015). The findings of a meta-analysis suggested that short LTL is associated with a high risk of coronary heart disease (Haycock et al., 2014). Likewise, a recent meta-analysis reported that LTL is inversely related to the risk of atrial fibrillation, especially in men (Zheng et al., 2022). Furthermore, aberrant LTL is associated with a variety of diseases, such as major depressive disorder (Pisanu et al., 2020), female fertility (Michaeli et al., 2022), complications of type 2 diabetes mellitus (Testa et al., 2011), Barrett’s esophagus (Risques et al., 2007), and non-obstructive azoospermia (Yang et al., 2018). Hence, screening risk factors leading to the aberrant LTL in the early stage is vital to disease prevention.

Obesity is a severe public health problem worldwide and the main triggering factor for diseases (Adom et al., 2017; Cheng et al., 2020; Wang et al., 2021). Substantial evidence has shown that obesity is associated with abnormal change in LTL (Buxton et al., 2011; Zhang et al., 2021). A negative correlation between obesity and LTL was reported in a recent meta-analysis (Zadeh et al., 2021), and the finding of a recent observational study has shown that obesity is inversely associated with LTL in Asian children (Ooi et al., 2021). Moreover, a meta-analysis including 63 studies and involving 119,439 populations revealed a negative correlation between obesity and LTL (Mundstock et al., 2015). Despite that considerable evidence suggests that obesity is negatively associated with LTL, a large heterogenicity has been found in different studies, and the causal relationship between obesity and LTL remains unclear. Therefore, we propose a hypothesis that obesity may be a potential causal risk factor to cause LTL changes.

Mendelian randomization (MR) study is a powerful epidemiological approach for investigating the causality between exposure and outcome by using genetic variants as instrumental variables (Burgess, Butterworth & Thompson, 2013). Given that the allocation of genetic variants is a randomization process and is not affected by extraneous postnatal factors (Emdin, Khera & Kathiresan, 2017), MR controlling residual confounding factors is the same as a randomized controlled trial (RCT) (Davies, Holmes & Davey Smith, 2018; Ference, Holmes & Smith, 2021). In the present work, a two-sample MR study was conducted to investigate the causal relationship between obesity and LTL. The inverse-variance weighted (IVW) method was used as the primary analysis algorithm to assess the potential causation. We are seeing the perniciousness of obesity in human health. Clarifying obesity’s potential causal influence on LTL is beneficial in drawing up strategies for preventing diseases. As far as we know, this is the first MR study investigating the causality between obesity-related traits and LTL. Nevertheless, more molecular experiments are necessary to investigate further the potential mechanism for the effect of obesity on LTL.

## Materials & Methods

### Study design

The summary-level data of obesity-related traits, including body mass index (BMI), whole body fat mass, leg fat mass (right), leg fat mass (left), arm fat mass (right), arm fat mass (left), and trunk fat mass were extracted from the IEU Open GWAS database (https://gwas.mrcieu.ac.uk/). The summary statistical data of GWAS associated with LTL were also obtained from the IEU Open GWAS database. The data were from large sample studies and were used in conducting the two-sample MR investigation and exploring the causal relationship between obesity-related traits and LTL.

### Assumptions of MR study

**Figure 1:**
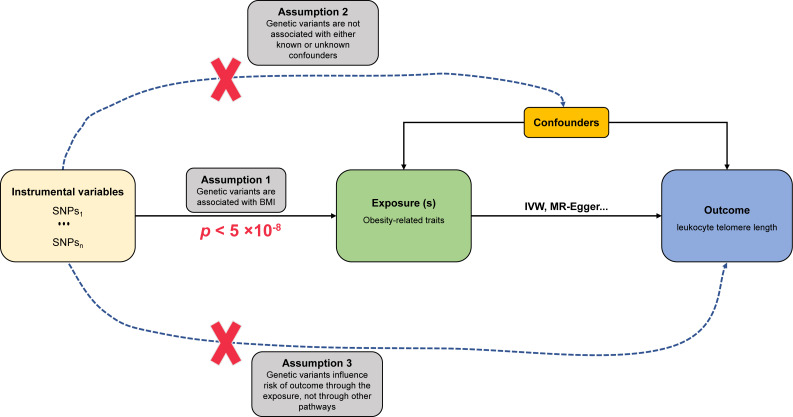
Schematic diagram of MR investigating the causal relationship between obesity and LTL. The instrumental variable (IV) assumptions: (1) the IVs must be strongly associated with obesity-related traits (*p* < 5 × 10^−8^); (2) the IVs must not be correlated with any unmeasured confounders of obesity-related traits *vs.* LTL relationship; (3) the IVs should only affect the risk of LTL *via* obesity-related traits. SNPs, single-nucleotide polymorphisms; LTL, leukocyte telomere length; IVW, inverse-variance-weighted.

The fundamental assumptions and design of the MR investigation are shown in Fig. 1. (1) Relevance assumption: The genetic variants (instrumental variables) must be strongly correlated with obesity-related traits (exposure). (2) Independence assumption: no unpredictable confounders of the correlations between genetic variants and LTL (outcome) are present. (3) Exclusion restriction assumption: the genetic variants influence LTL only *via* obesity-related traits.

### Data sources

The GWAS summary statistical data of obesity-related traits were obtained from European populations. The information included whole body fat mass with 330,762 participants, leg fat mass (right) with 331,293 populations, leg fat mass (left) with 331,275 populations, arm fat mass (right) with 331,226 individuals, arm fat mass (left) with 331,164 people, and trunk fat mass with 331,093 volunteers; all above obesity-related traits were obtained from UK Biobank cohort of the Neale lab. Single-nucleotide polymorphisms (SNPs) associated with obesity-related traits were identified as instrumental variables based on these parameters: *p* < 5 × 10^−8^ as a genome-wide statistical significance, independence among SNPs in linkage disequilibrium (*r*^2^ < 0.001; clump window, 10,000 kb). The F statistic was used in assessing the instrumental variables’ power in the MR and the computational method described by a previous study (Pierce, Ahsan & Vanderweele, 2011). *F* statistic > 10 was defined as the minimum required threshold. The GWAS summary data associated with LTL from European ancestry (472,174 individuals) were downloaded from the IEU Open GWAS database. All participants in the obesity-related trait research projects were not screened for the LTL cohort.

### Statistical analysis

The IVW was used as a primary analysis algorithm in estimating the causal effect size of obesity-related traits on LTL (Burgess, Butterworth & Thompson, 2013). The MR-Egger (Bowden, Davey Smith & Burgess, 2015), maximum likelihood (Xue, Shen & Pan, 2021), MR-pleiotropy residual sum outlier (MR-PRESSO) (Verbanck et al., 2018), and robust adjusted profile score (MR-RAPS) (Zhao et al., 2020) algorithms were utilized in validating the reliability and robustness for the causal relationship between obesity traits and LTL. An available online tool (https://shiny.cnsgenomics.com/mRnd/) (Brion, Shakhbazov & Visscher, 2013) was used to calculate the statistical power of MR analysis, and a power greater than 80% was deemed as a good value. The directionality that obesity-related traits cause LTL was verified *via* the MR Steiger test (Hemani, Tilling & Davey Smith, 2017). *p* < 0.05 indicated statistical significance.

### Sensitivity analysis

The heterogeneity of SNPs was inspected *via* the IVW method and MR-Egger regression. MR-PRESSO, MR-Egger, and IVW approaches were used in identifying and removing outliers. The MR-Egger algorithms were utilized to detect potential pleiotropy. A single SNP’s influence on the total effect of IVW was evaluated through leave-one-out permutation analysis.

All MR analyses were conducted using TwoSampleMR (version 0.5.6) and MRPRESSO packages in R software (version 4.1.2; R Core Team, 2021).

## Results

### Causality between BMI and LTL

**Figure 2:**
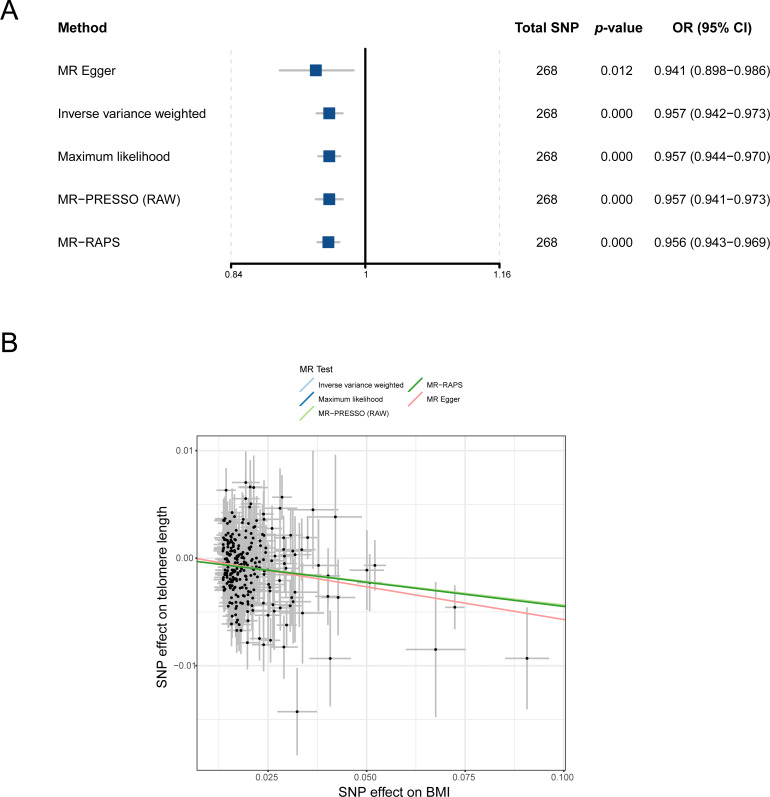
MR analysis of the causal relationship between BMI and LTL. (A) Forest plot for MR analysis visualization; (B) scatter plots of the correlation between BMI and LTL. MR, Mendelian randomization; SNP, single-nucleotide polymorphism; BMI, body mass index; MR-PRESSO, MR-pleiotropy residual sum outlier; MR-RAPS, robust adjusted profile score; OR, odds ratio; CI, confidence interval.

To understand the causal relationship between obesity and LTL preliminarily, we analyzed the effect of BMI on LTL by using the IVW method in the two-sample MR study. After outliers were removed, all 268 independent SNPs associated with BMI were included in the MR for the computation of the pool effect size of BMI on LTL (File S1). The result of the IVW approach showed that a genetically determined 1-SD increase in BMI was correlated with decreased LTL, and the odds ratio (OR) was 0.957 (95% confidence interval [CI] = 0.942–0.973, *p* = 1.17E−07; Table 1 and Fig. 2A). In the analysis, the F statistic of the SNPs was 61.0, and the statistical power was 100%, indicating the absence of weak-instrument bias and high credibility, respectively (Table 1).

**Table 1:** MR Results of obesity-related traits’ influence on LTL.

**Exposure**	**Method**	**No. of SNPs**	***p***	**OR (95% CI)**	***p*_-het_**	***p*_-intercept_**	***F* statistic**	**Power**
BMI	MR Egger	268	0.012	0.941 (0.898–0.986)	4.48E−07	0.448		
	**Inverse-variance-weighted**	268	1.17E−07	0.957 (0.942–0.973)			61.00	100%
	Maximum likelihood	268	2.07E−10	0.957 (0.944–0.970)				
	MR-PRESSO (RAW)	268	2.45E−07	0.957 (0.941–0.973)				
	MR-RAPS	268	6.70E−11	0.956 (0.943–0.969)				
Whole body fat mass	MR Egger	247	0.040	0.942 (0.890–0.997)	3.44E−12	0.782		
	**Inverse-variance-weighted**	247	3.69E−08	0.949 (0.932–0.967)			60.00	100%
	Maximum likelihood	247	1.03E−12	0.949 (0.936–0.963)				
	MR-PRESSO (RAW)	247	9.26E−08	0.949 (0.931–0.968)				
	MR-RAPS	247	1.34E−13	0.948 (0.934–0.961)				
Leg fat mass (right)	MR Egger	242	0.057249543	0.931 (0.865–1.002)	2.36E−15	0.976		
	**Inverse-variance-weighted**	242	6.98E−09	0.932 (0.910–0.954)			58.90	100%
	Maximum likelihood	242	2.38E−15	0.931 (0.914–0.947)				
	MR-PRESSO (RAW)	242	2.17E−08	0.932 (0.909–0.955)				
	MR-RAPS	242	4.44E−16	0.930 (0.913–0.946)				
Leg fat mass (left)	MR Egger	245	0.013970688	0.918 (0.858–0.982)	8.34E−10	0.683		
	**Inverse-variance-weighted**	245	3.79E−10	0.931 (0.910–0.952)			59.20	100%
	Maximum likelihood	245	2.85E−15	0.931 (0.914–0.947)				
	MR-PRESSO (RAW)	245	1.70E−09	0.931 (0.909–0.953)				
	MR-RAPS	245	4.44E−16	0.929 (0.912–0.945)				
Arm fat mass (right)	MR Egger	240	0.114083631	0.959 (0.910–1.010)	7.85E−08	0.889		
	**Inverse-variance-weighted**	240	3.67E−07	0.955 (0.939–0.972)			60.72	100%
	Maximum likelihood	240	4.59E−10	0.955 (0.942–0.969)				
	MR-PRESSO (RAW)	240	7.41E−07	0.955 (0.938–0.973)				
	MR-RAPS	240	1.40E−10	0.954 (0.941–0.968)				
Arm fat mass (left)	MR Egger	236	0.018611648	0.939 (0.892–0.989)	2.44E−07	0.689		
	**Inverse-variance-weighted**	236	4.63E−09	0.949 (0.932–0.965)			61.07	100%
	Maximum likelihood	236	9.11E−13	0.949 (0.935–0.962)				
	MR-PRESSO (RAW)	236	1.56E−08	0.949 (0.931–0.966)				
	MR-RAPS	236	2.18E−13	0.947 (0.934–0.961)				
Trunk fat mass	MR Egger	247	0.034146424	0.942 (0.891–0.995)	3.55E−10	0.376	58.78	100%
	**Inverse-variance-weighted**	247	5.53E−05	0.964 (0.947–0.981)				
	Maximum likelihood	247	3.43E−07	0.964 (0.951–0.978)				
	MR-PRESSO (RAW)	247	7.37E−05	0.964 (0.947–0.982)				
	MR-RAPS	247	1.26E−07	0.963 (0.950–0.977)				

**Notes.**

BMI body mass index OR odds ratio CI confidence interval *p*-het *p*-value for heterogeneity using Cochran *Q* test *p*-intercept *p*-value for MR-Egger intercept MR-PRESSO Mendelian randomization-pleiotropy residual sum outlier MR-RAPS robust adjusted profile score SNP single-nucleotide polymorphism

A series of sensitivity analysis approaches was used in verifying the robustness and reliability of the above result. First, as shown in Fig. 2B, four methods (MR-Egger, maximum likelihood, MR-PRESSO, and MR-RAPS) consistently exhibited causal direction from BMI to LTL. The result suggested that the causality from BMI to LTL was stable. Second, the results from Cochran’s Q test in the IVW model (*p*_-het_ = 4.76E−07) and MR-Egger model (*p*_-het_ = 4.48E−07) indicated heterogeneity among the SNPs, which may have been caused by the random allocation of alleles. Third, the statistical result of the MR-Egger intercepts indicated no directional pleiotropy in the MR analysis (*p*_-intercept_ = 0.448). Fourth, leave-one-out analysis showed that no single SNP significantly influenced the causal association between BMI and LTL (*p* < 0.05; File S2). Finally, statistical evidence from the MR Steiger test suggested that BMI influencing LTL was a correct causal direction (*p* < 0.001; Table 1).

### Causality between six body fat indexes and LTL

Previous studies reported some bias when only BMI was used as an indicator for measuring obesity and estimating the correlation between obesity and diseases (Nimptsch, Konigorski & Pischon, 2019; Piche, Tchernof & Despres, 2020; Vecchie et al., 2018) because BMI cannot reflect body fat distribution. Therefore, to avoid the bias of causality, six body fat indexes (whole body fat mass, right leg fat mass, left leg fat mass, right arm fat mass, left arm fat mass, and trunk fat mass) were used to confirm the casual assumption of obesity impact on LTL. After outliers were deleted, 247, 242, 245, 240, 236, and 247 independently available SNPs were associated with whole body fat mass, right leg fat mass, left leg fat mass, right arm fat mass, left arm fat mass, and trunk fat mass, respectively (File S1). The SNPs were then utilized in proving the genetically predicted causality between obesity and LTL. The result of the IVW algorithm indicated that enhanced body fat indexes are causally associated with a decrease in LTL. The effect size of six body fat indexes’ influences on LTL were as follows: whole body fat mass: OR = 0.949 (95% CI [0.932–0.967]; *p* = 3.69E−08), right leg fat mass: OR = 0.932 (95% CI [0.910–0.954]; *p* = 6.98E−09), left leg fat mass: OR = 0.931 (95% CI [0.910–0.952]; *p* = 3.79E−10), right arm fat mass: OR = 0.955 (95% CI [0.939–0.972]; *p* = 3.67E−07), left arm fat mass: OR = 0.949 (95% CI [0.932–0.965]; *p* = 4.63E−09), and trunk fat mass: OR = 0.964 (95% CI [0.947–0.981]; *p* = 5.53E−05; Table 1). In the analyses, the F statistics of the SNPs were larger than 10.0 (range: 58.8–61.1), indicating the absence of potential weak instrument bias, and all statistical power rates were approximately 100%, indicating high credibility (Table 1).

**Figure 3:**
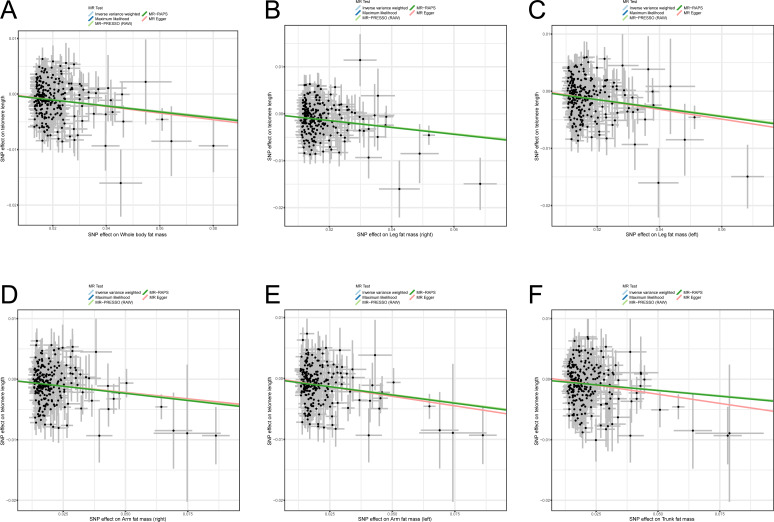
Scatter plots of the correlation between six body fat indexes and LTL. (A) Whole body fat mass; (B) right leg fat mass; (C) left leg fat mass; (D) right arm fat mass; (E) left arm fat mass; (F) Trunk fat mass. MR, Mendelian randomization; SNP, single-nucleotide polymorphism; MR-PRESSO, MR-pleiotropy residual sum outlier; MR-RAPS, robust adjusted profile score.

Likewise, sensitivity analysis methods were used in testing the robustness and reliability of the above result. First, MR-Egger, maximum likelihood, MR-PRESSO, and MR-RAPS were utilized in validating the stability of the causal hypothesis. The results uniformly showed that the causality between per body fat index and LTL was stable (Figs. 3A–3F). We analyzed the heterogeneity of per body fat index-related SNPs by using Cochran’s Q test in the IVW model and the MR-Egger model. The results suggested heterogeneity among SNPs (all *p*_-het_ < 0.05; Table 1). Furthermore, uncorrelated horizontal pleiotropy was detected *via* the MR-Egger method, and the result showed that the MR analyses had no uncorrelated horizontal pleiotropy (all *p*_-intercept_ < 0.01; Table 1). Moreover, we used the iterative leave-one-out analysis method to determine whether a single SNP significantly modifies the pool effect value of the IVW. The result indicated that no single SNP significantly disrupted the combined effect of IVW (all *p* < 0.05; File S2). Finally, the causal hypothesis of the six body fat indexes’ influences on LTL was examined using the MR Steiger algorithm. The results suggested that the six body fat indexes affecting LTL was the correct causal direction (all *p* < 0.001).

## Discussion

In the present work, we first used the extensive sample GWAS summary data of European populations to investigate the causal relationship between obesity-related features and LTL comprehensively. Results show that genetically predicted higher BMI, whole body fat mass, right leg fat mass, left leg fat mass, right arm fat mass, left arm fat mass, and trunk fat mass were all causally associated with a lower LTL. These findings are the first to prove that obesity was a key causal risk factor that led to a decrease in LTL.

Previous studies have shown that aberrant LTL can increase the risk of many diseases (Codd et al., 2013; Purdue-Smithe et al., 2021; Sanders & Newman, 2013). Thus, protecting LTL may be an essential way of preventing illness. Obesity is a highly prevalent disease and seriously harms people (Conway & Rene, 2004). Although substantial evidence supports that obesity is inversely correlated with LTL, the strength of the correlation is uneven, and the causality between obesity and LTL remains unexplained. This situation might be associated with the use of different indicators in measuring obesity. A meta-analysis included 16 original studies to investigate the correlation between BMI and LTL and found a negative association between BMI and LTL in adults (Muezzinler, Zaineddin & Brenner, 2014). Similarly, a cross-sectional study with 1000 participants found that BMI and LTL has a negative association (Muezzinler et al., 2016). A negative association between BMI and LTL was reported in a cross-sectional study with 35,096 individuals (Williams et al., 2016). A recent large meta-analysis including 87 studies and involving 146,114 individuals investigated the association between BMI and LTL in different age categories, gender, and ethnicity; a negative correlation between BMI and LTL was found, especially in younger participants (Gielen et al., 2018). In the current study, the standardized data of LTL was used to analyze the causal relationship between BMI and LTL and found BMI’s impact on LTL was a correct causal direction (OR = 0.957 [95% CI [0.942–0.973]], *p* = 1.17E−07). The finding initially illustrated the causal relationship between obesity and LTL. The statistical evidence of several sensitivity analyses confirmed that our result was stable and reliable.

Despite that BMI remains to be a primary indicator for assessing obesity in clinical works, bias occurs when obesity is measured with BMI alone because BMI does not reflect body fat distribution (Antonopoulos et al., 2016). Body fat mass may be a better and more direct indicator for describing obesity (Kesztyus, Lampl & Kesztyus, 2021; Lee & Giovannucci, 2019). A previous observational study with a small sample (45 women) reported body fat mass was negatively correlated with LTL (Shin & Lee, 2016). Similarly, the finding of an observational study including 145 healthy term-born infants indicated that body fat mass was negatively associated with LTL (De Fluiter et al., 2021). Although these observational studies suggested a negative association between fat mass and LTL, the evidence was limited because they only used a single fat mass to estimate the causality between obesity and LTL. Therefore, to accurately assess the causal relationship between obesity and LTL, we used six body fat mass indexes (whole body fat mass, right leg fat mass, left leg fat mass, right arm fat mass, left arm fat mass, and trunk fat mass) to evaluate obesity’s influence on LTL. We found that the indexes were all inversely associated with LTL. These results again proved that the causal direction from obesity to LTL was correct. The present study overcomes the limitations of traditional observational studies that neither well control for unmeasured confounding nor prove causality between exposure and outcome (Davies, Holmes & Davey Smith, 2018).

## Limitations

The current study possesses some shortcomings. First, obesity was divided into four types: metabolically healthy obesity, metabolically obese normal weight phenotype, normal weight obese syndrome, and sarcopenic obesity (Vecchie et al., 2018). Whether our result applies to all types is unknown. Second, we failed to achieve stratification analysis according to age and gender. Third, genetic variation is only one of the factors causing exposure changes, and many more remain to be further explored, such as environmental and epigenetic factors. Finally, given that the MR study was conducted in European ancestry, whether it can be popularized in non-European ancestry need to be investigated further.

## Conclusions

In conclusion, using the extensive GWAS summary data, we implemented two-sample MR investigations to examine the causal relationship between BMI and LTL. We identified potential causal effects of several obesity-related traits on LTL. Our results suggested that genetically predicted obesity is inversely associated with LTL. In addition, given that the lifelong adverse effects of obesity on LTL are due to genetic variants, our findings may be useful in formulating persistent strategies for maintaining LTL and promoting health.

##  Supplemental Information

10.7717/peerj.15085/supp-1 File S1 Details of the genetic variants (instrumental variables)

10.7717/peerj.15085/supp-2 File S2 Results of leave-one-out analysis

10.7717/peerj.15085/supp-3 File S3 The STROBE-MR checklist, RAW data, and R Code

## Additional Information and Declarations

### Data Availability

The following information was supplied regarding data availability: The STROBE-MR checklist, RAW data, and R code are available in the Supplemental Files.
